# Brain network construction and analysis for patients with mild cognitive impairment and Alzheimer's disease based on a highly‐available nodes approach

**DOI:** 10.1002/brb3.2027

**Published:** 2021-01-03

**Authors:** Xiaopan Zhang, Junhong Liu, Yuan Chen, Yanan Jin, Jingliang Cheng, for the Alzheimer’s Disease Neuroimaging Initiative

**Affiliations:** ^1^ Department of Magnetic Resonance Imaging The First Affiliated Hospital of Zhengzhou University Zhengzhou China

**Keywords:** Alzheimer's disease, mild cognitive impairment, network graph, resting‐state functional magnetic resonance imaging, support vector machine

## Abstract

**Introduction:**

Using brain network and graph theory methods to analyze the Alzheimer's disease (AD) and mild cognitive impairment (MCI) abnormal brain function is more and more popular. Plenty of potential methods have been proposed, but the representative signal of each brain region in these methods remains poor performance.

**Methods:**

We propose a highly‐available nodes approach for constructing brain network of patients with MCI and AD. With resting‐state functional magnetic resonance imaging (rs‐fMRI) data of 84 AD subjects, 81 MCI subjects, and 82 normal control (NC) subjects from the Alzheimer's Disease Neuroimaging Initiative Database, we construct connected weighted brain networks based on the different sparsity and minimum spanning tree. Support Vector Machine of Radial Basis Function kernel was selected as classifier.

**Results:**

Accuracies of 74.09% and 77.58% in classification of MCI and AD from NC, respectively. We also performed a hub node analysis and found 18 significant brain regions were identified as hub nodes.

**Conclusions:**

The findings of this study provide insights for helping understanding the progress of the AD. The proposed method highlights the effectively representative time series of brain regions of rs‐fMRI data for construction and topology analysis brain network.

## Graphical abstract

A highly‐available nodes algorithm for constructing brain network of patients with MCI and AD was proposed. The brain networks were constructed by using the Minimum spanning tree and the different sparsity. With using a hub node analysis, 18 significant brain regions were identified as hub nodes.

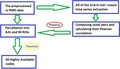

## 1 INTRODUCTION

Patients with Alzheimer's disease (AD) or mild cognitive impairment (MCI) show a decline in memory and cognitive functions than healthy people (Watt & Karl, 2017). Studies have shown that the brains of AD or MCI patients changed before the clinical symptoms appear early (Celone Willment et al., 2006; Greicius et al., 2004). Recently, many studies revealed the differences of functional connectivity of AD brain regions based on resting‐state functional magnetic resonance imaging (rs‐fMRI) (Ali et al., 2017; Sharaev et al., 2016; Yu et al., 2020). Specially, it is more and more popular that using brain network and graph theory methods to analyze the AD and MCI abnormal brain function (Lei et al., 2020; Wang, Shen, et al., 2018).

So far, several groups have developed methods to investigate changes in functional brain organization in patients with AD and MCI. Liu had used a method, which is based on the partial correlations and indirect dependencies between each pair of brain regions to calculate the abnormal patterns of AD brain (Liu, Zhang, Yan, et al., 2012). Zhang had constructed cortical diffusivity networks using graph theoretical approach. He defined connection as statistical associations in gray matter elevated mean diffusivity (MD) value between every two brain areas, and then, they constructed a symmetric connection matrix to analyze the AD and MCI abnormal brain function (Zhang et al., 2015). Cui and Liu had developed a Multivariate Predictors model, which extracted multiple features from different modalities of data. This model can explore an optimal set of predictors in AD abnormal brain (Cui et al., 2011). Ali Khazaee had used directed graph measures to identify alteration of brain network in MCI and AD. They drew a conclusion that patients with MCI and AD may experience disappearing some hub regions during disease progression (Ali et al., 2017). Si had provided a brain network model for studying the mechanism underlying the development of AD and MCI. In this model, they had adopted not only anatomical distance but also network topology, such as topology‐based link prediction methods and naïve Bayes classifiers (Si et al., 2019). These methods effectively used information of brain network topology, and they had obtained rational results at that time. Simultaneously, for a large proportion of these methods, the first step was to segment the rs‐fMRI data into different regions by using some kind of partition template, and then signals of each region were averaged to generate a representative signal for each region. They calculated the correlation of different regions based on these representative voxels. However, the averaged signal is not sufficient to reveal complex topological information of the brain region. The connection network that construct based on averaged signals lacks a deeper interaction between the brain regions and the topological differences between patient group and normal control (NC) group.

In order to address the above problem, we propose a highly‐available nodes approach for constructing brain network of patients with MCI and AD. The connected weighted brain networks at the different sparsity and minimum spanning tree (MST) were constructed base on this method. To date, many studies employed the classification of function brain networks to investigate alterations in MCI and AD brain regions (Ali et al., 2017; Yue et al., 2011; Wee et al., 2012). The performance for classification of patients with AD and MCI from NC subjects was selected to evaluate the preponderance of our algorithm and conventional algorithm. In addition, studies have shown that identifying the brain regions associated with neurodegeneration was correlated the complex interactive information in the network (Celone Willment et al., 2006; Liu, Zhang, Bai, et al., 2012). We hypothesized that identifying the hub nodes of AD, MCI, and NC brain regions would achieve better results, because the node information is more effective, in which the brain networks were constructed base on our method.

## 2 MATERIALS AND METHODS

Data used in the preparation of this article were obtained from the Alzheimer's Disease Neuroimaging Initiative (ADNI) Database (http://adni.loni.usc.edu/). A large proportion of the information in the ADNI are magnetic resonance imaging (MRI), other biological markers, and clinical and neuropsychological assessment about MCI and AD. For up‐to‐date information, see www.adni‐info.org. ADNI researchers collect several types of data from study volunteers throughout their participation in the study, using a standard set of protocols and procedures to eliminate inconsistencies. At the time of enrollment for data collection, subjects gave written informed consent and completed questionnaires approved by each participating site's Institutional Review Board.

The data processing procedures were approved by the First Affiliated Hospital of Zhengzhou University Scientific research and clinical trial ethics committee (No: 2018‐KY‐88).

### 2.1 Subjects

We selected 247 subjects from the ADNI Database, 124 males and 123 females, aged from 47 to 82 years:. 84 patients with AD, 81 patients with MCI, and 82 NC subjects. The dementia severity of subjects was evaluated by the Mini‐Mental State Examination (Folstein et al., 1975) and the Clinical Dementia Rating (Morris et al., 1997). For the details, see Table 1.

**TABLE 1:** Demographic characteristics of subjects

	AD	MCI	NC
Number	84	81	82
Male/female	41/43	42/39	41/41
Age range (year)	(51–82)	(49–77)	(47–73)
Age (Mean ± *SD*)	74.17 ± 5.28	70.31 ± 3.18	69.88 ± 6.13
MMSE score (Mean ± *SD*)	16.28 ± 3.27	27.85 ± 2.42	28.97 ± 6.48
CDR score (Mean ± *SD*)	0.93 ± 0.28	0.48 ± 0.07	0.02 ± 0.17

Abbreviations: AD, Alzheimer's disease; CDR, Clinical Dementia Rating; MCI, mild cognitive impairment; MMSE, Mini‐Mental State Examination; NC, normal control.

### 2.2 Data acquisition and preprocessing

Functional and structural MRI scans were acquired from three tesla (3T) scanner. Functional MRI images were acquired with repetition time (TR) = 3,000 ms, echo time (TE) = 30 ms, slice thickness = 3.0 mm and flip angle (FA) = 80°. Structural MRI images were obtained using a 3‐dimensional high‐resolution sagittal T1W. Parameters: TR = 600 ms, TE = 11 ms and slice thickness = 0.9 mm.

All preprocessing steps were performed with Statistical Parametric Mapping (SPM8) software (https://www.fil.ion.ucl.ac.uk/spm/) (Litvak et al., 2011) and Data Processing Assistant for Resting‐State fMRI (DPARSF) toolbox (http://www.restfmri.net) (Yan & Zang, 2010). The first 10 volumes of the functional images’ session were discarded to allow for equilibrations of the magnetic field and slice‐timing correction to the last slice. All the remaining volumes were realigned for head movement compensation correct using the least‐squares minimization (Li et al., 2020). Without subjects had head rotations greater than 1° or head movements exceeding 2 mm on any axis. Then, the imaging data were standardized based on the Montreal Neurological Institute space and resampled at 3 mm × 3 mm × 3 mm (Ashburner & Friston, 1999). Finally, the imaging data were smoothed by using a Gaussian filter with full width at half maximum (FWHM) of 4 mm (Guo et al., 2019). Temporal band‐pass filtering (0.01–0.08 Hz) was performed to reduce the effects of low‐frequency drifts and high‐frequency noise (Sharaev et al., 2016).

### 2.3 Highly‐available node calculation

**FIGURE 1:**
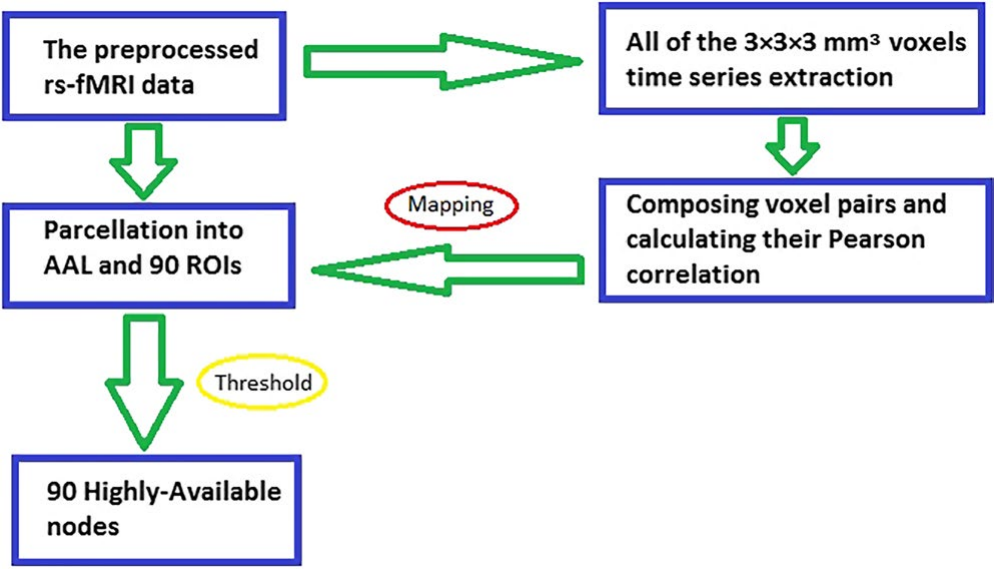
Flow chart representation of the highly‐available node calculation

The overall procedure of the highly‐available node calculation is shown in Figure 1. At first, we extracted all of the 3 × 3 × 3 mm^3^ voxel time series from the preprocessed rs‐fMRI data, and then, let these voxels compose pairs randomly and calculated the Pearson correlation of every voxel pair by the following algorithm.

(1) $$r_{\mathrm{ij}}=\frac{\sum \left[x_{i}\left(t\right)‐X_{i}\right]\left[x_{j}\left(t\right)‐X_{j}\right]}{\sqrt{\sum {\left[x_{i}\left(t\right)‐X_{i}\right]}^{2}\sum {\left[x_{j}\left(t\right)‐X_{j}\right]}^{2}}}$$

In Equation (1), the $x_{i}$ and $x_{j}$ are the BOLD signal of voxel *i* and voxel *j* at time *t*, the $X_{i}$ and $X_{j}$ represent the average value of voxel *i* and voxel *j*.

In this paper, a nondirectional connected Pearson correlation was used to make for the judgment of whether it was correlative between voxels (or brain regions). The Pearson *r* of auto correlation and negative correlation was deemed to zero.

Secondly, the rs‐fMRI data were segmented into different regions base on some anatomically divisional template. In this study, 90 regions of interest (ROIs), each hemisphere 45 ROIs were obtained by using the anatomical automatic labeling template (Tzourio‐Mazoyer et al., 2002). Removing the voxel pairs in each ROIs, the rest of the voxel pairs are between different ROIs. Based on a threshold of Pearson *r* to remove those voxel pairs with low scores, we speculated the suitable threshold of Pearson *r* according to the following criteria:

Ⅰ, under the threshold of Pearson *r*, for each voxel, if there is at least one voxel in different ROI connect with it, it is defined as an effective voxel.
Ⅱ, for every ROI, there is at least one effective voxel being included in it.

Finally, each ROI was regarded as a highly‐available node. In order to constrain each pair of voxels from different ROIs in a randomly way, we calculated the representative time series of each node by the following algorithm.

(2) $$A=\frac{1}{N}\sum N_{k}A_{k}$$

In Equation (2), two voxels of each voxel pair were coming from different ROIs. The *N* is the number of voxel pairs of all voxels in this ROI; the *N_k_* is the number of voxel pairs of voxel *k*; the *A_k_* is the time series of voxel *k*.

### 2.4 Brain network construction

For every subject, with 90 brain ROIs for the node, build full connected weighted brain networks. We calculated the Pearson correlation between each pair of ROIs, as node connection strength in the brain network (Schindler et al., 2008).

Although full connected weighted brain networks theoretical analyses are helpful for understanding disease mechanisms, it may get more incorrect results due to their redundant connective information. To deal with this problem, we used MST method and based on sparsity method to simplify the brain networks. The MST is acyclic and connects all nodes in the original graph (Mieghem & Magdalena, 2005). The method of MST can remove redundant connection and keep the network core structure simultaneously, and it does not affect the overall analysis of the network. In this study, the MST method was applied based on the weighted networks with Kruskal's algorithm (Choi & Lee, 2013). Suppose WN=(V,E) is a connection network with *n* nodes, in which the set of nodes is *V* and the set of edges is *E*. The first step of constructing the MST, the weights of all links are ranked in an ascending order and a subgraph with only *n* nodes and empty edge set is constructed (Tewarie et al., 2015). Then, an edge with the largest weight is selected from *E*, it is added to the subgraph if the two nodes of this edge belong to different trees. The following edges with the largest weight are added to the subgraph in the same way, until all nodes are connected in the subgraph that consists of n‐1 edges (Tewarie et al., 2015; Wang, Miao, et al., 2018).

To ensure that the resulting graphs metrics were accordant, they would be composed of same numbers of edges, and the weights of the brain functional network must to be filtered by a threshold (Liu, Zhang, Yan, et al., 2012). The sparsity was generally used as the threshold metrics for all the correlation matrices to simplify the brain networks (Achard & Bullmore, 2007; Scheltens, 2007). Because there is no gold standard for threshold selection, we simplified each original graph over a wide range of sparsity (6%≤S ≤ 36%), and based on 6% step length in this interval.

### 2.5 Feature selection and classification

In traditional brain function connection network analysis, the following graph measures had been calculated generally degree, betweenness centrality (BC), node strength, clustering coefficient, range coefficient, transitivity, and assortativity etc. (Guo et al., 2018; Shaoqiang et al., 2020). In this study, weighted brain networks were constructed to study the deeper mutual information between brain regions. The degree, BC, and clustering coefficient have good performances of node aggregation degree in the weighted network (Bloznelis, 2013; Sporns et al., 2007) and can well reflect the prevalence of each node and the situation around in brain network (Rubinov & Sporns, 2010). The degree *k_i_* is the number of connections of the node *i* (Liu & Tian, 2014). The BC is the number of shortest paths through a node. The clustering coefficient is fraction of triangles around an individual node and is equivalent to the fraction of the node's neighbors (Rubinov & Sporns, 2010; Watts & Strogatz, 1998). The degree, BC, and local clustering coefficient of each region were calculated for every weighted brain network. The features were selected by using the two‐sample *t* test method, and the functional connections which the p values of any two of these three measures were less than 0.05 were selected as feature. The Bonferroni test was used as calibration method in the significance tests (Bland & Altman, 1995). In this study, the significance level was set at *p* < .05.

Support Vector Machine (SVM) is a popular, powerful, supervised machine learning method for classification (Burges, 1998; Chang & Lin, 2011). SVM method usually constructs linear classification boundaries by using a kernel function in high dimensional spaces, and it is a common method for features classification in brain networks. In this study, a SVM that kernel function is Radial Basis Function (RBF) was selected (Chang & Lin, 2011). For evaluating the performance of classification, the accuracy, specificity, and sensitivity are often used in literature. Here, a 10‐fold cross‐validation was used to robust classification. The benchmark dataset was randomly divided into 10 subsets. One subset was selected as a test set, and the other subsets composed the corresponding training set, repeat that 10 times. For evaluating the performance of classification, accuracy, specificity, and sensitivity are often used in literature (Ali et al., 2017).

### 2.6 Identifying hub nodes

Hub nodes play a central role in overall organization of the brain network, and they are important brain regions that underpin numerous aspects of complex cognitive function (Ali et al., 2017; van den Heuvel & Sporns, 2013). In order to explore the changes of local brain areas during the pathological process of AD, we identified the hub nodes of function connection network by calculating the BC of nodes. We measured the normalized betweenness $b_{i}$ of the node *i* by the following equation. Nodes with high values of $b_{i}$ were identified as the hubs of the brain networks.

(3) $$b_{i}=\frac{B_{i}}{\left\langle B\right\rangle }$$

In Equation (3) (He et al., 2008), *B_i_* is the BC of the node *i*; ⟨B⟩ is the average BC of the network.

## 3 RESULTS

### 3.1 Constructing function connection brain network

**FIGURE 2:**
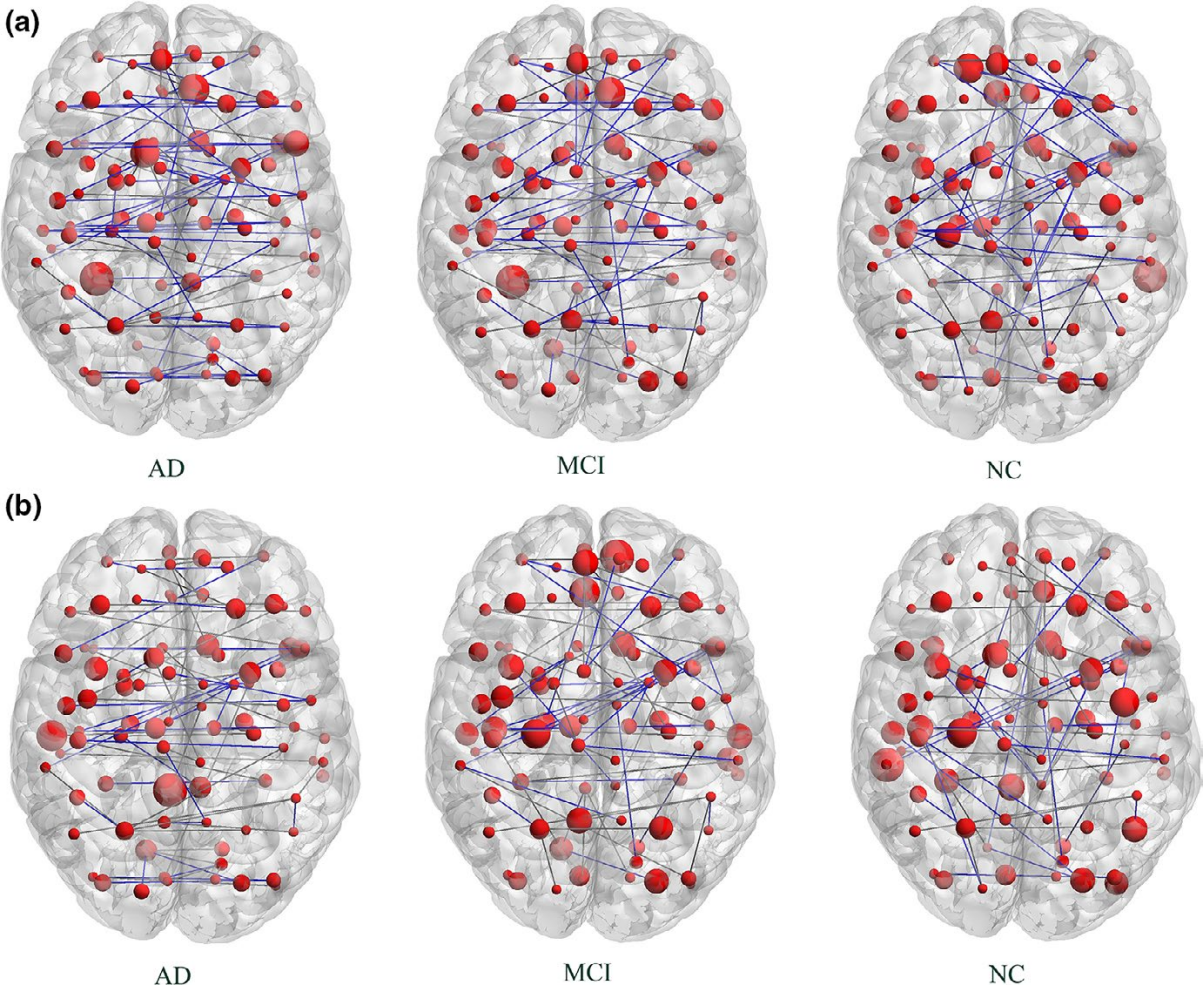
Network structures of average MST for three groups. The size of nodes represents the connective edge number of brain regions, bigger size said the number of connective edges about this ROI was more, and smaller size said the edges was fewer. The color of edge indicates the path length between nodes, blue is a short length, black represents a long length. (a) The time series of each ROI was calculated by the method of highly‐available node calculation. (b) The time series of each ROI was calculated by the conventional algorithm

In this study, the MST of all subjects were constructed, each MST contained 90 nodes and 89 edges. To further show the distinctions of MST within three groups, we constructed the MST based on the average adjacency matrix of each group respectively (shown in Figure 2). In this paper, graph theoretical visualization were performed by BrainNet viewer software (https://www.nitrc.org/projects/bnv/) (Mingrui et al., 2013). In addition, the function connection brain networks were constructed at the sparsity of 6%, 12%, 18%, 24%, 30%, and 36% orderly.

### 3.2 Classification of patients with AD and MCI from NC

To examine the performance of our algorithm for classification of patients with AD and MCI from NC subjects, we contrasted it with conventional algorithm on the same dataset. For conventional algorithm, the representative time series of each ROI was calculated by averaging the time series of voxels within each of 90 regions. In different function connection network, selecting SVM with RBF kernel as classifier, performance of these two algorithms was compared using 10‐fold cross‐validation (shown in Table 2). A better performance was achieved using our algorithm than conventional method. It is noteworthy that our algorithm achieved an accuracy of 74.09% in classification of MCI from NC and 77.58% in classification of AD from NC in function connection networks at the sparsity of 12%.

**TABLE 2:** Performance of classification using different function connection network

Network	Method	Accuracy (%)		Specificity (%)		Sensitivity (%)	
		MCI	AD	MCI	AD	MCI	AD
MST	This paper	72.11	76.32	80.73	79.28	86.40	89.52
	Conventional algorithm	63.85	69.08	74.62	71.85	78.33	81.09
Sparsity of 6%	This paper	69.36	71.54	72.83	77.42	67.20	66.71
	Conventional algorithm	59.92	62.16	60.70	73.52	53.28	61.44
Sparsity of 12%	This paper	74.09	77.58	85.38	81.07	85.00	88.13
	Conventional algorithm	70.43	70.99	81.27	73.31	76.52	82.40
Sparsity of 18%	This paper	70.82	72.07	80.10	79.28	79.91	81.07
	Conventional algorithm	65.33	69.76	73.05	69.48	70.37	78.59
Sparsity of 24%	This paper	68.00	67.46	70.30	75.93	73.04	79.82
	Conventional algorithm	60.53	61.47	64.86	69.57	70.48	69.55
Sparsity of 30%	This paper	62.77	64.28	72.00	79.88	73.92	77.19
	Conventional algorithm	55.92	59.28	67.93	72.01	66.00	71.52
Sparsity of 36%	This paper	59.01	61.36	65.73	77.24	65.17	68.77
	Conventional algorithm	52.40	54.71	59.97	69.38	62.44	61.06

Abbreviations: AD, Alzheimer's disease; MCI, mild cognitive impairment; MST, minimum spanning tree.

Several other algorithms had also been applied in classification of patients with MCI and AD from NC. To prove the classification performance of our algorithm, we contrasted it with Feng Li's Robust Deep Model (Li et al., 2015) and Ali Khazaee's directed graph measure (Ali et al., 2017) on the same dataset. In order to perform a fair comparison with different methods, all methods used exactly the same SVM with RBF kernel as classifier, 10‐fold cross‐validation, and MST network. Particularly, the classification performances of different methods were clearly shown in Table 3, which indicate that our method had the best classification performance.

**TABLE 3:** Compared with different methods for performance of classification

Method	Accuracy (%)		Specificity (%)		Sensitivity (%)	
	MCI	AD	MCI	AD	MCI	AD
This paper	72.11	76.32	80.73	79.28	86.40	89.52
Feng Li's Robust Deep Model	70.93	72.41	75.02	76.37	81.94	85.06
Ali Khazaee's directed graph measure	71.85	72.90	74.55	79.07	80.52	87.33

Abbreviations: AD, Alzheimer's disease; MCI, mild cognitive impairment.

### 3.3 Brain regions related to the cognitive status related to AD and MCI

As reported in Section 3.2, the connection networks at the sparsity of 12%, we got the best classification of AD and MCI from NC. The brain regions with large values in $b_{i}$ > 1.7 were identified as the hubs of connection networks (He et al., 2008; Liu, Zhang, Yan, et al., 2012). In this study, we constructed the brain network at the sparsity of 12%, and the hub nodes were identified with $b_{i}$ > 1.7. For our method, 18 ROIs were identified as hub nodes in the three groups (shown in Table 4). For the conventional algorithm, 15 ROIs were identified as hub nodes in the three groups (shown in Table 5).

**TABLE 4:** Regions showing high betweenness in brain networks by using the highly‐available nodes approach

Name of brain regions	Normalized betweenness,$b_{i}$		
	AD	MCI	NC
Right hippocampus	0.326	**1.970**	**4.842**
Left middle frontal	**3.831**	1.512	0.327
Right middle frontal	**4.271**	1.176	0.315
Left superior temporal gyrus	0.173	**2.847**	1.252
Left posterior cingulate gyrus	0.378	0.832	**2.311**
Left lingual	**3.436**	**1.772**	0.580
Right olfactory cortex	**2.732**	0.743	1.310
Left orbital superior frontal gyrus	**2.169**	0.352	1.601
Right rolandic operculum	0.073	**1.874**	**2.501**
Left middle frontal orbital	**3.809**	1.630	0.427
Left amygdala	0.134	**2.878**	**3.180**
Right posterior cingulate gyrus	1.483	0.532	**2.392**
Right middle temporal	1.431	0.589	**2.875**
Right parahippocampal	**3.359**	0.247	**2.926**
Left middle temporal	1.007	0.086	**1.975**
Right amygdala	**3.642**	1.576	0.722
Left hippocampus	1.282	0.145	**1.709**
Left calcarine	1.661	**3.387**	0.425

Note

The bold font represents the ROI being with $b_{i}$ > 1.7 in the corresponding group.

Abbreviations: AD, Alzheimer's disease; MCI, mild cognitive impairment; NC, normal control.

**TABLE 5:** Regions showing high betweenness in brain networks by using the conventional algorithm

Name of brain regions	Normalized betweenness,$b_{i}$		
	AD	MCI	NC
Left middle frontal	**3.712**	1.624	0.319
Left superior temporal gyrus	0.278	1.342	**2.612**
Left posterior cingulate gyrus	**1.935**	0.109	**2.783**
Left lingual	**3.063**	0.760	1.512
Right olfactory cortex	**3.025**	0.691	0.408
Left calcarine	**2.726**	1.080	0.319
Right putamen	1.201	**2.931**	0.213
Right posterior cingulate gyrus	0.075	**1.804**	**2.192**
Right middle temporal	1.022	0.118	**1.783**
Left orbital superior frontal gyrus	**2.027**	1.605	0.267
Right parahippocampal	**3.518**	0.728	1.652
Left middle frontal orbital	**1.928**	**2.705**	0.137
Right amygdala	0.367	1.158	**2.870**
Right hippocampus	0.218	1.452	**3.734**
Left hippocampus	0.957	0.031	**1.995**

Note

The bold font represents the ROI being with $b_{i}$ > 1.7 in the corresponding group.

Abbreviations: AD, Alzheimer's disease; MCI, mild cognitive impairment; NC, normal control.

**FIGURE 3:**
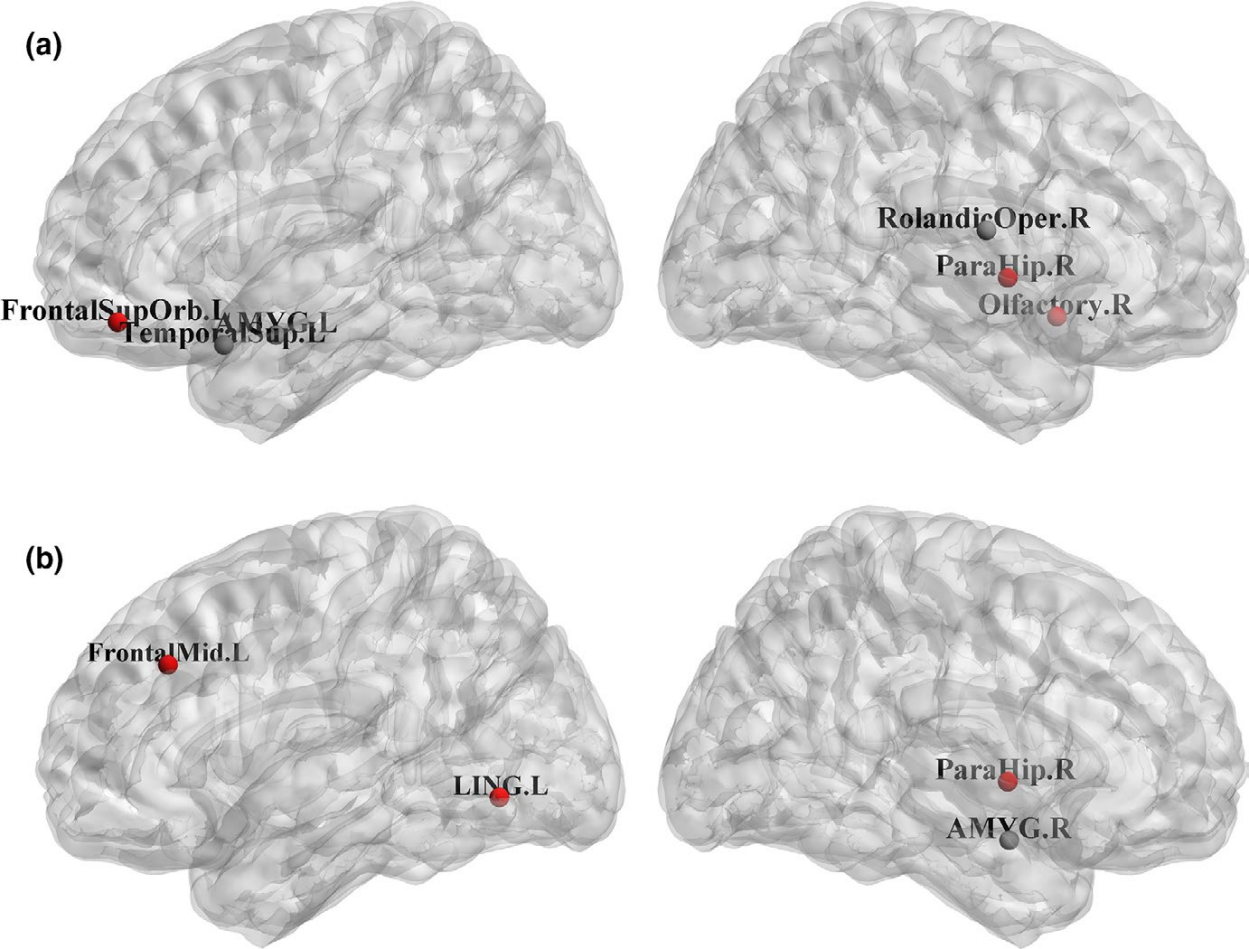
Brain regions showing abnormal nodal centrality in AD subjects compared with MCI subjects. The color of nodes represents the decreased (black) or increased (red) nodal centrality in AD subjects compared with MCI subjects. (a) The time series of each ROI was calculated by the method of highly‐available node calculation. (b) The time series of each ROI was calculated by the conventional algorithm. Label: Left hippocampus (HIP.L), Right hippocampus (HIP.R), Left middle frontal (FrontalMid.L), Right middle frontal (FrontalMid.R), Left superior temporal (TemporalSup.L), Right posterior cingulate gyrus (CingulumPost.R), Left posterior cingulate gyrus (CingulumPost.L), Left lingual (LING.L), Right olfactory cortex (Olfactory.R), Right rolandic operculum (RolandicOper.R), Left calcarine (CAL.L), Left amygdala (AMYG.L), Right middle temporal (TemporalMid.R), Right parahippocampal (ParaHip.R), Left middle frontal orbital (FrontalMidOrb.L), Left middle temporal (TemporalMid.L), Left orbital superior frontal gyrus (FrontalSupOrb.L), Right amygdala (AMYG.R), Right putamen (Putamen.R), Left middle frontal orbita (FrontalMidOrb.L)

**FIGURE 4:**
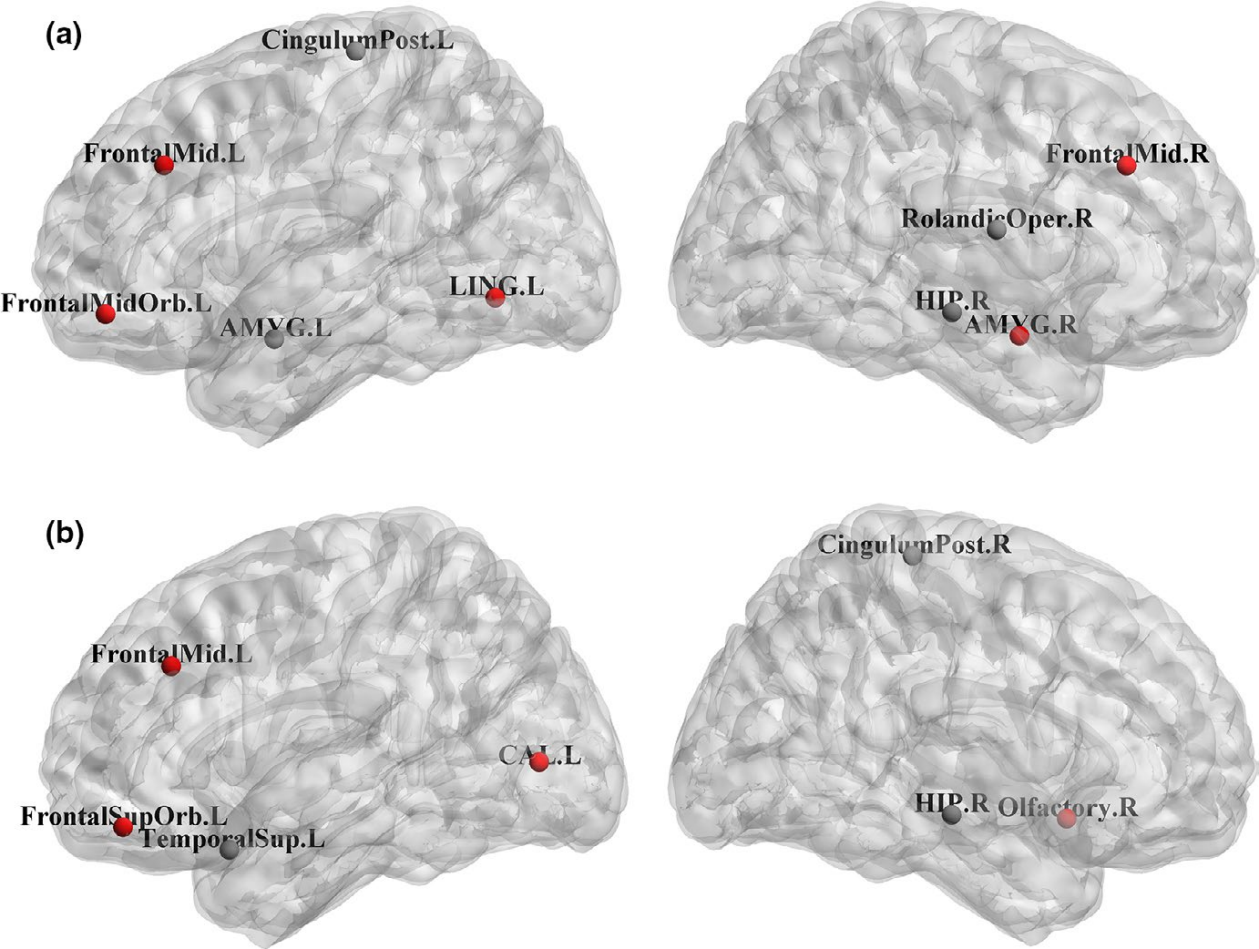
Brain regions showing abnormal nodal centrality in AD subjects compared with NC subjects. The color of nodes represents the decreased (black) or increased (red) nodal centrality in AD subjects compared with NC subjects. (a) The time series of each ROI was calculated by the method of highly‐available node calculation. (b) The time series of each ROI was calculated by the conventional algorithm. The label details were shown in Figure 3

**FIGURE 5:**
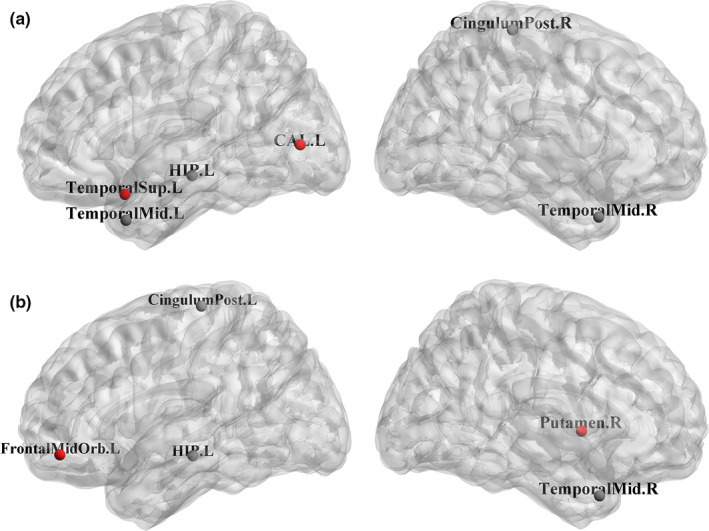
Brain regions showing abnormal nodal centrality in MCI subjects compared with NC subjects. The color of nodes represents the decreased (black) or increased (red) nodal centrality in MCI subjects compared with NC subjects. (a) The time series of each ROI was calculated by the method of highly‐available node calculation. (b) The time series of each ROI was calculated by the conventional algorithm. The label details were shown in Figure 3

Finally, we examined the changes of the normalized betweenness between different groups (AD and MCI, AD and NC, MCI and NC) severally. The ROIs of the significant changes (*p* < .05) between different groups were shown as follows. For our method, compared with MCI, AD subjects showed the BC decreases in the brain regions of the left superior temporal gyrus, the right rolandic operculum, and the left amygdala, while the BC increases were in the brain regions of the right olfactory cortex, the left orbital superior frontal gyrus, and the right parahippocampal (shown in Figure 3). Compared with NC, AD subjects showed the BC decreases in the brain regions of the right hippocampus, the left posterior cingulate gyrus, the right rolandic operculum, and the left amygdala, while the BC increases were in the brain regions of the left middle frontal, the right middle frontal, the left lingual, the left middle frontal orbital, and the right amygdala (shown in Figure 4). Compared with NC, MCI subjects showed BC decreases in the brain regions of the right posterior cingulate gyrus, the left middle temporal, the right middle temporal, and the left hippocampus, while the BC increases were in the brain regions of the left superior temporal gyrus and the left calcarine (shown in Figure 5). For conventional algorithm, compared with MCI, AD subjects showed the BC decreases in the brain area of the right amygdala, while the BC increases were in the brain regions of the left middle frontal, the left lingual, and the right parahippocampal (shown in Figure 3). Compared with NC, AD subjects showed the BC decreases in the brain regions of the right hippocampus, the left superior temporal gyrus, and the right posterior cingulate gyrus, while the BC increases were in the brain regions of the left middle frontal, the right olfactory cortex, left calcarine, and the left orbital superior frontal gyrus (shown in Figure 4). Compared with NC, MCI subjects showed the BC decreases in the brain regions of the left posterior cingulate gyrus, the right middle temporal, and the left hippocampus, while the BC increases were in the brain regions of the right putamen and the left middle frontal orbital (shown in Figure 5).

## 4 DISCUSSION

Here, we have presented a highly‐available nodes approach for constructing and analyzing brain network of patients with MCI and AD. Based on this method, we respectively constructed the weighted rs‐fMRI brain networks for each subject on a database of total 247 subjects (the subjects detail shown in Table 1). With SVM of RBF, kernel was selected as classifier, and accuracies of 74.09% and 77.58% were achieved for classification of MCI and AD from NC, respectively. In order to demonstrate the ability of our method for classification of patients with AD and MCI from NC subjects, we contrasted it with conventional algorithm on the same dataset. Our method achieved a better performance (shown in Table 2).

In addition, we analyzed the property of connection network for each subject, and 18 significant brain regions were identified as hub nodes by using our method. Comparing with the conventional algorithm, four brain regions were more obtained. Remarkably, the brain regions of the right middle frontal and the left middle temporal gyrus had been reported in the studies by Ali Khazaee (Ali et al., 2017). In the studies of Liu, the brain regions of the right rolandic operculum and the left amygdala were reported as regions with significant different nodal centrality between MCI patients and AD patients (Liu, Zhang, Yan, et al., 2012). So, the result of these hub brain regions was dependable.

What's more, using network graphs to study clinical problems can provide useful insights for helping understanding the progress of the disease. In this research, we examined the changes of the brain regions nodal centrality between different groups (AD and MCI, AD and NC, MCI and NC) severally. We obtain that the brain regions of the left superior temporal gyrus, the left amygdala, and the left middle frontal showed significant difference in at least two groups of AD ‐ MCI, AD – NC, and MCI ‐ NC. Previous studies suggest that ceruloplasmin level and gene expression changes at the superior temporal gyrus were associated with aging and AD (Connor et al., 1993; Horesh et al., 2011). The neuropathological changes in the amygdala may be linked to the conversions from the MCI to AD (Gallo et al., 2010; Liu et al., 2010). A recent study found that the centrality of the right middle frontal was decreased in AD patients (Guo et al., 2016). The brain regions showing different nodal centrality in AD and MCI reflect the brain functional transform in AD and MCI.

## 5 CONCLUSION

In this article, we have proposed an approach for constructing and analyzing brain network of patients with MCI and AD. Comparing with the conventional algorithm, it achieved a better performance in classification of MCI and AD from NC. In addition, with analyzing the nodal centrality of the brain networks in AD, MCI, and NC, 18 significant brain regions that identify as hub nodes were obtained. In a word, the highly‐available nodes approach provided the representative time series of brain area effectively and facilitated the algorithm of the brain network topology analysis to perform a precise level.

## CONFLICTS OF INTEREST

The authors declare that they have no competing interests exist.

## AUTHOR CONTRIBUTIONS

Xiaopan Zhang designed the study and drafted the manuscript. Xiaopan Zhang, Junhong Liu, and Yuan Chen performed the data collection and analyses. Yanan Jin and Jingliang Cheng provided guidance to the research question and technical advice on statistical analyses. All authors approved the final manuscript prior to submission.

### Peer Review

The peer review history for this article is available at https://publons.com/publon/10.1002/brb3.2027.

## Data Availability

The data that support the findings of this study are available from the Alzheimer's Disease Neuroimaging Initiative (ADNI) Database. Restrictions apply to the availability of these data, which were used under license for this study. Data are available at http://adni.loni.usc.edu/ with the permission of the ADNI Data and Publications Committee.
